# Supplementary figures and images for: Cell surface localisation of GPI-anchored receptors in Trypanosoma brucei
Source: eLife. 2026 May 19;14:RP107191. doi: 10.7554/eLife.107191 (PMC13186567; doi:10.7554/eLife.107191)

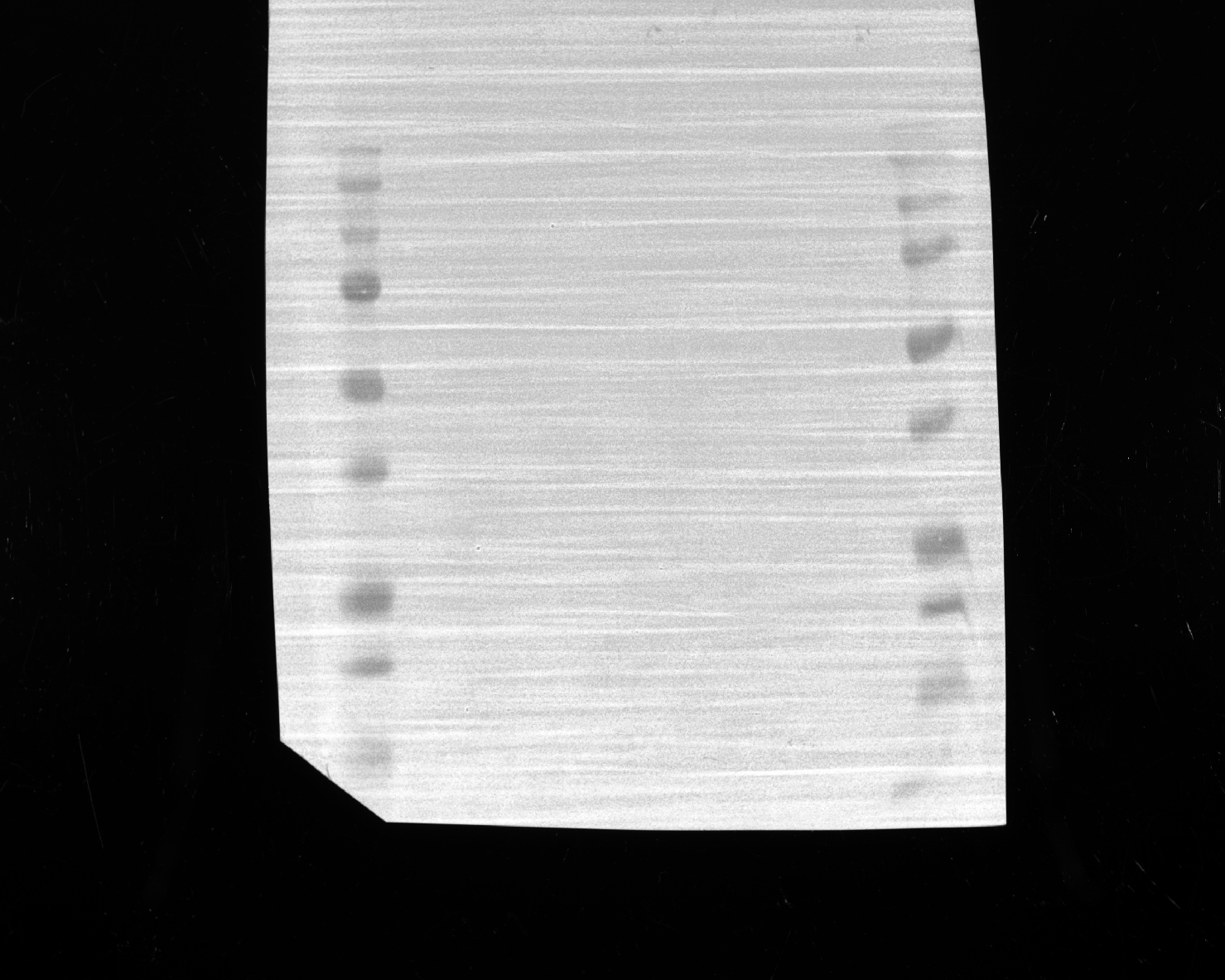

Supplement: Figure 1—source data 1. [file elife-107191-fig1-data1.zip › Figure 1_Source data 1/Figure 1_markers fro anti_TfR.jpg]

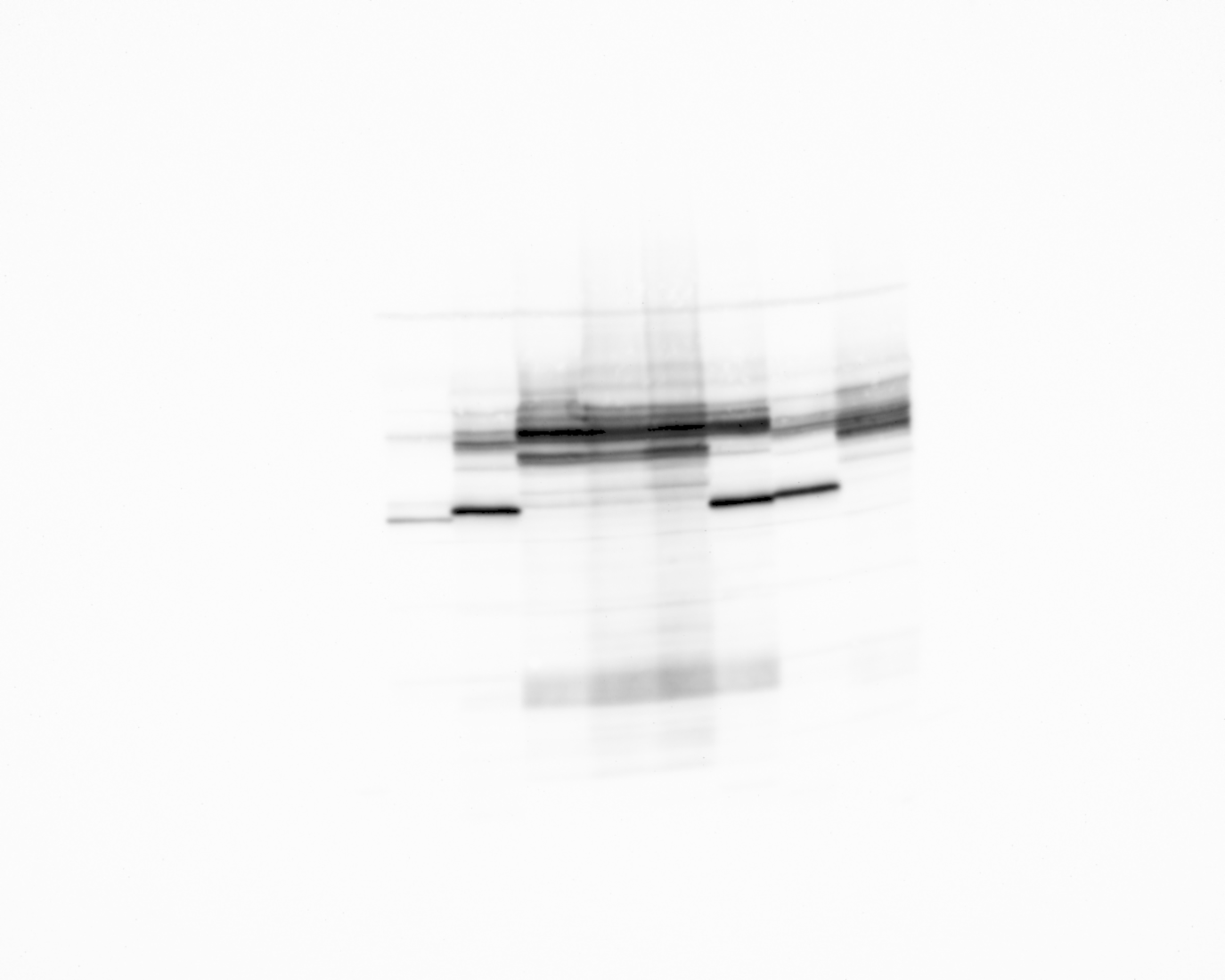

Supplement: Figure 1—source data 1. [file elife-107191-fig1-data1.zip › Figure 1_Source data 1/Figure 1_anti-TfR blot.tif]

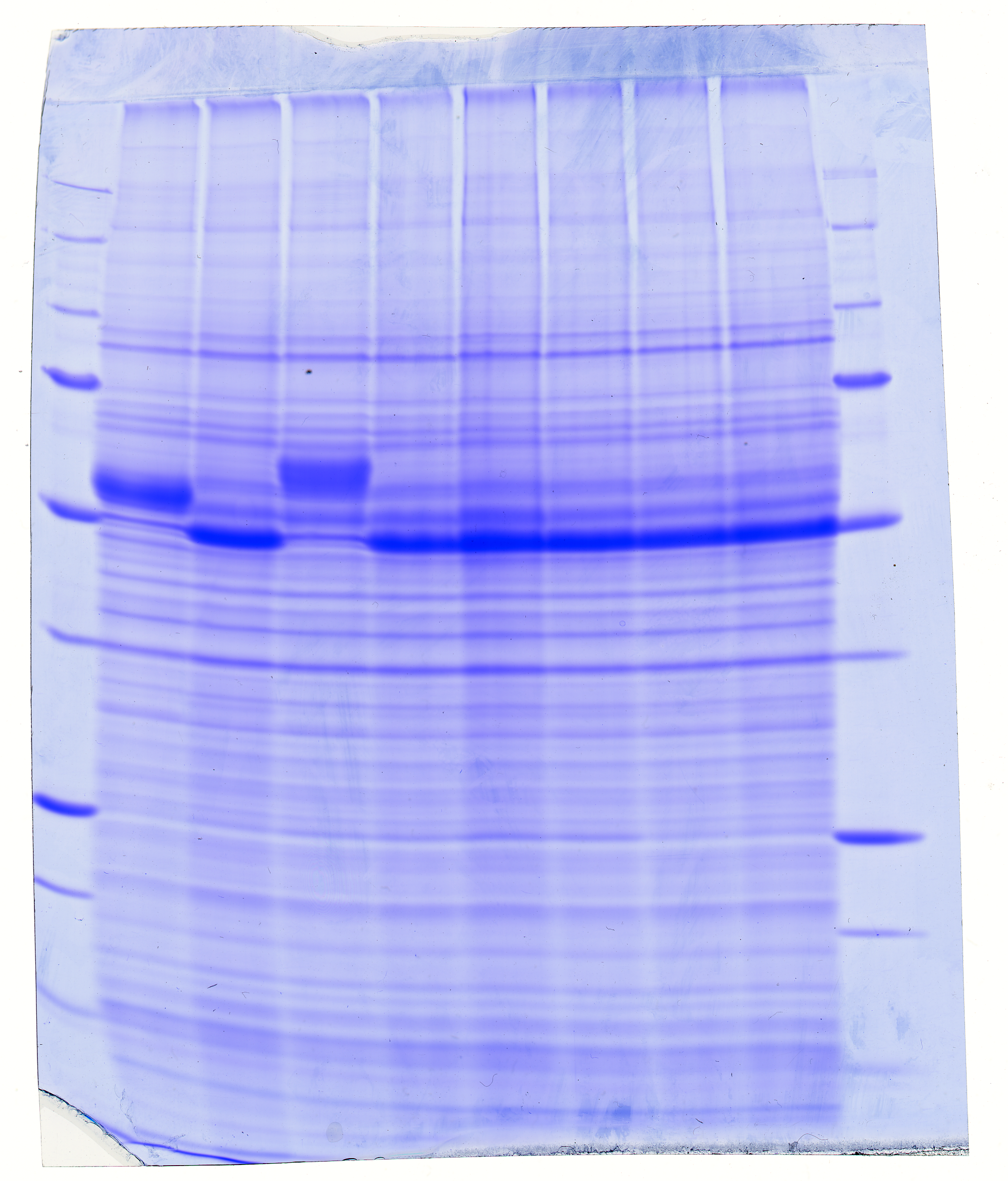

Supplement: Figure 1—source data 1. [file elife-107191-fig1-data1.zip › Figure 1_Source data 1/Figure-1-Coomassie-blue.tif]

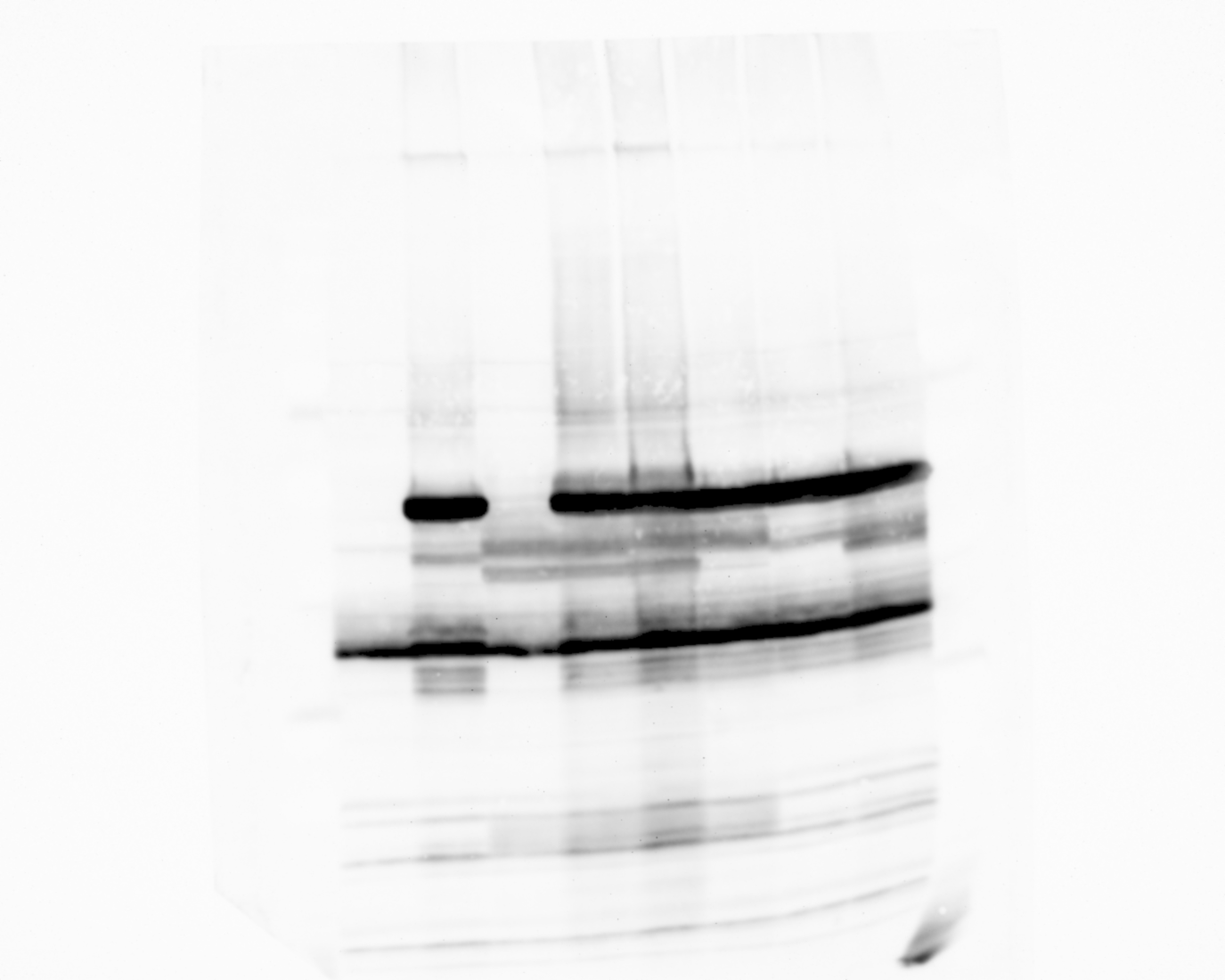

Supplement: Figure 1—source data 1. [file elife-107191-fig1-data1.zip › Figure 1_Source data 1/Figure 1_anti-VSG blot.tif]

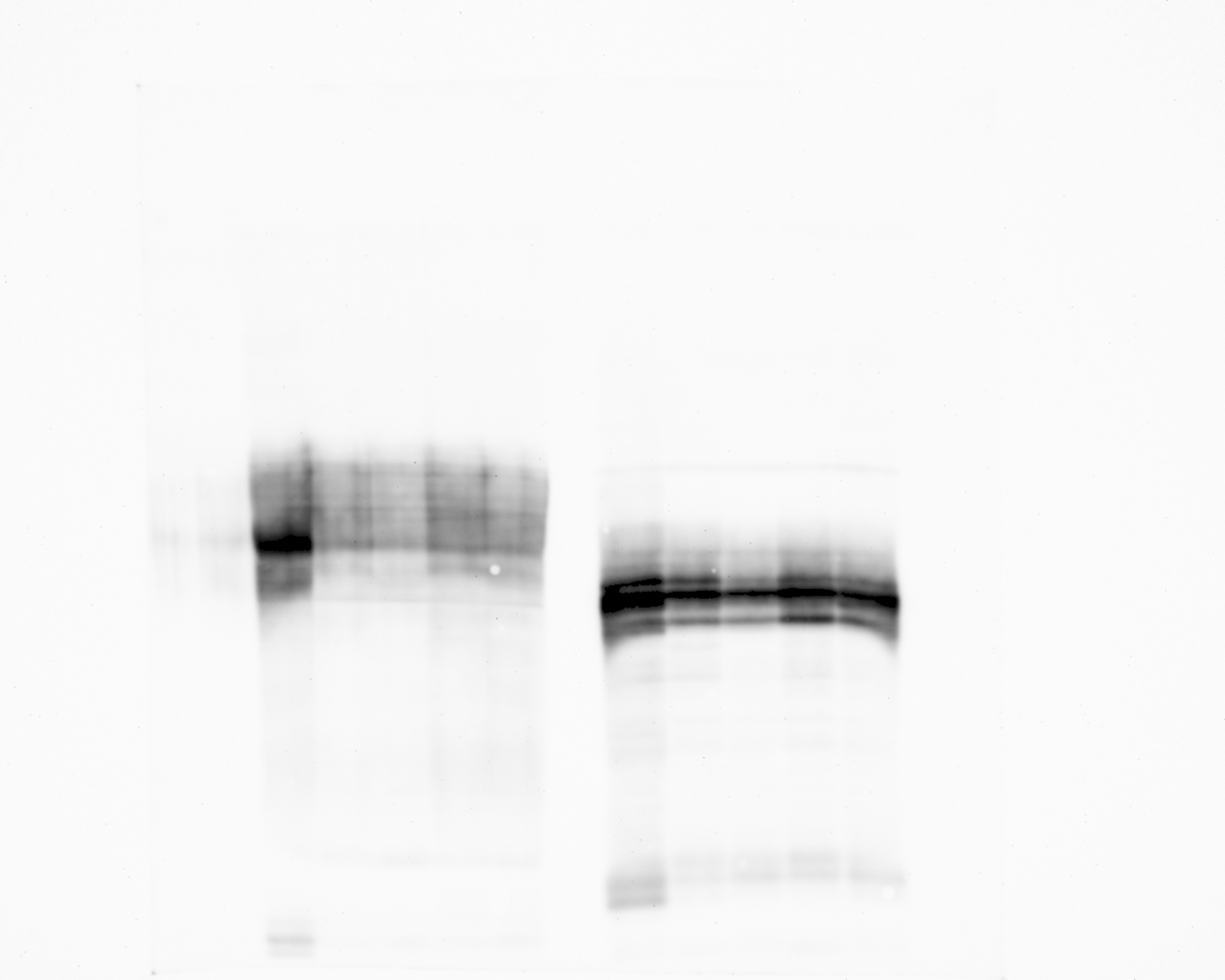

Supplement: Figure 3—figure supplement 4—source data 1. [file elife-107191-fig3-figsupp4-data1.zip › Figure S4_Source data 1/Figure S4_anti-TfR blot.tif]

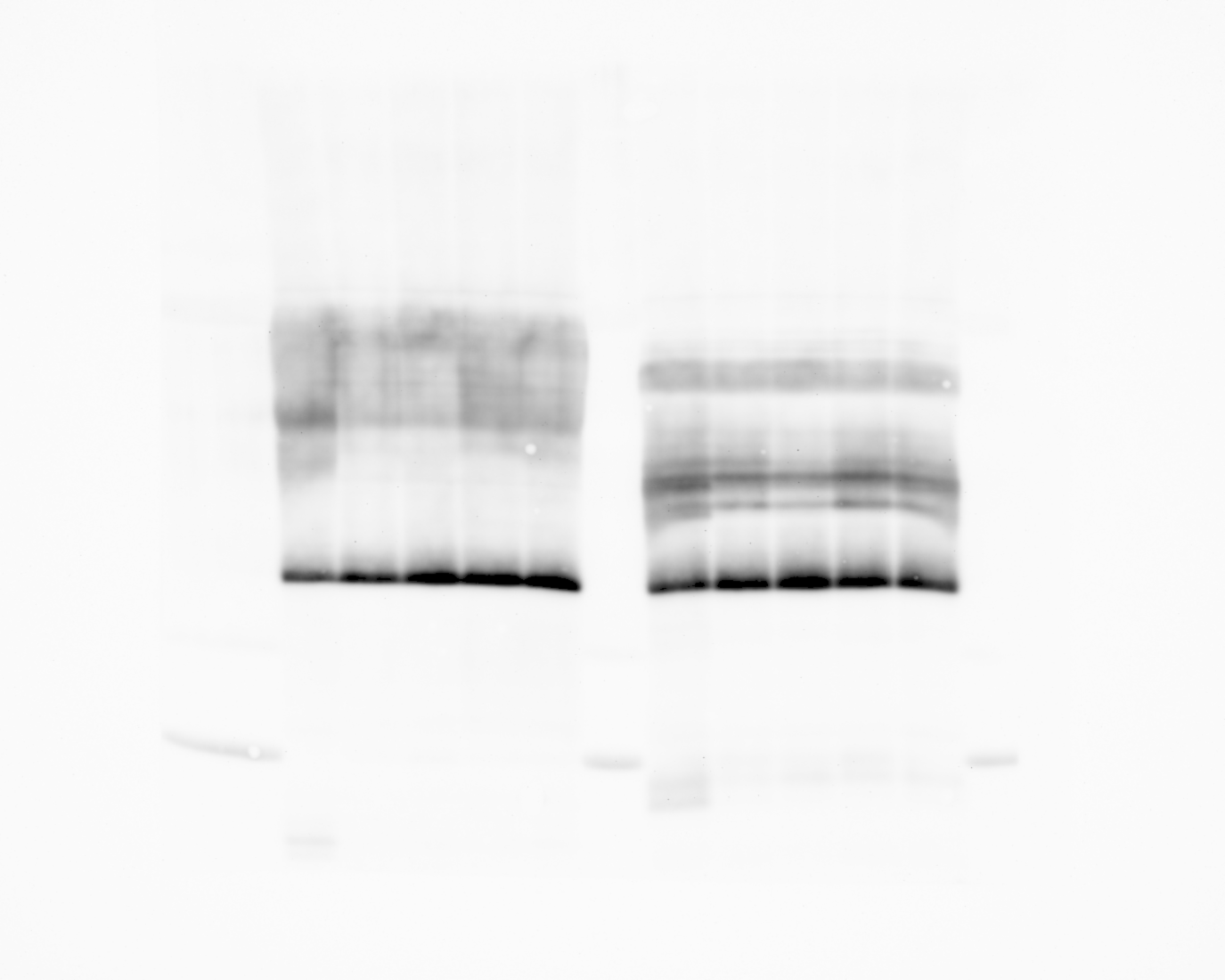

Supplement: Figure 3—figure supplement 4—source data 1. [file elife-107191-fig3-figsupp4-data1.zip › Figure S4_Source data 1/Figure S4_anti-SCD6 blot.tif]

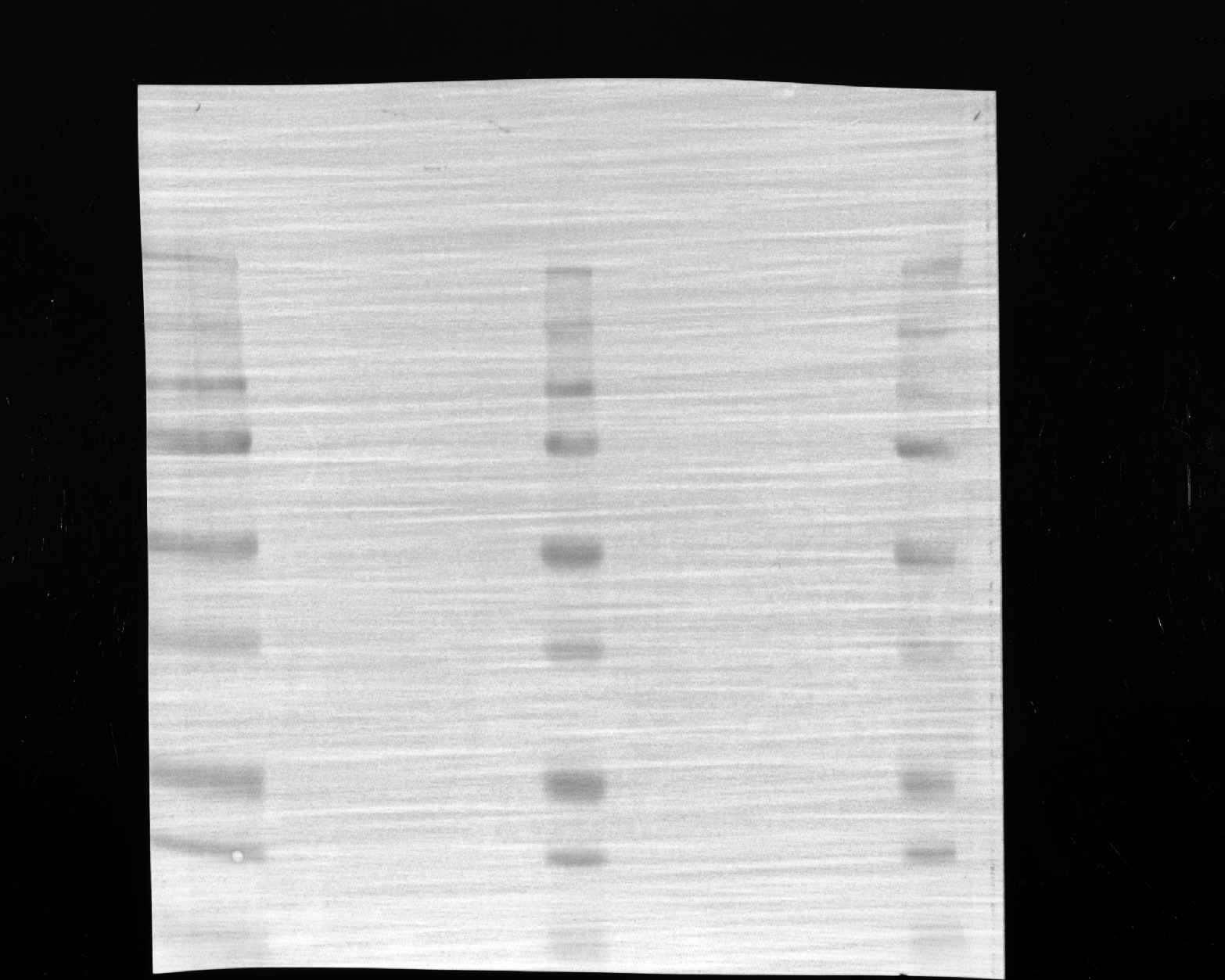

Supplement: Figure 3—figure supplement 4—source data 1. [file elife-107191-fig3-figsupp4-data1.zip › Figure S4_Source data 1/Figure S4_protein standards.tif]

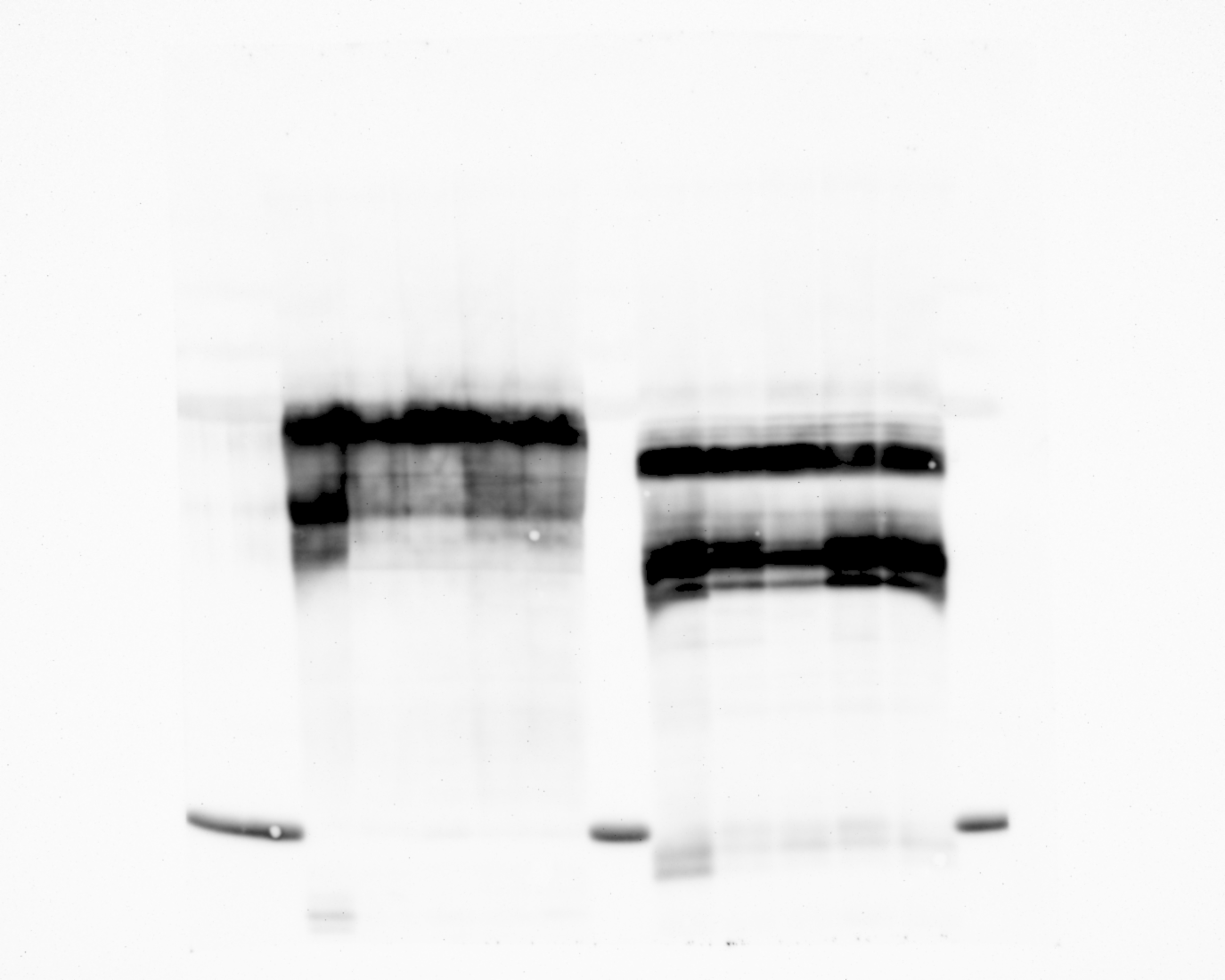

Supplement: Figure 3—figure supplement 4—source data 1. [file elife-107191-fig3-figsupp4-data1.zip › Figure S4_Source data 1/Figure S4_anti-ISG65 blot.tif]
