# Supplementary material for: Cell surface localisation of GPI-anchored receptors in Trypanosoma brucei
Source: eLife. 2026 May 19;14:RP107191. doi: 10.7554/eLife.107191 (PMC13186567; doi:10.7554/eLife.107191)
Supplement: Figure 1—source data 2. [file elife-107191-fig1-data2.zip › Figure 1_Source_data_2.pdf]

Colorimetric western blot protein standards-will overlay precisely with the anti-TfR blot

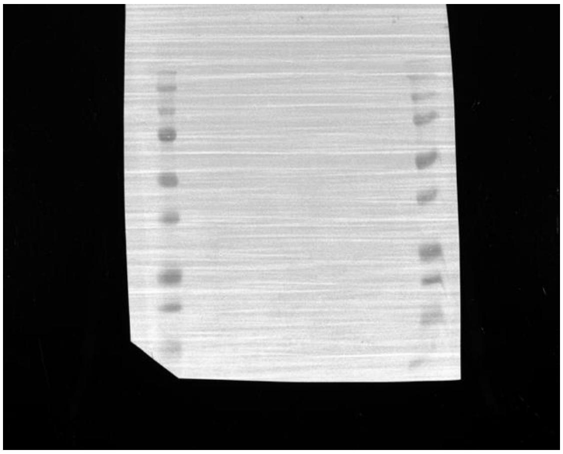

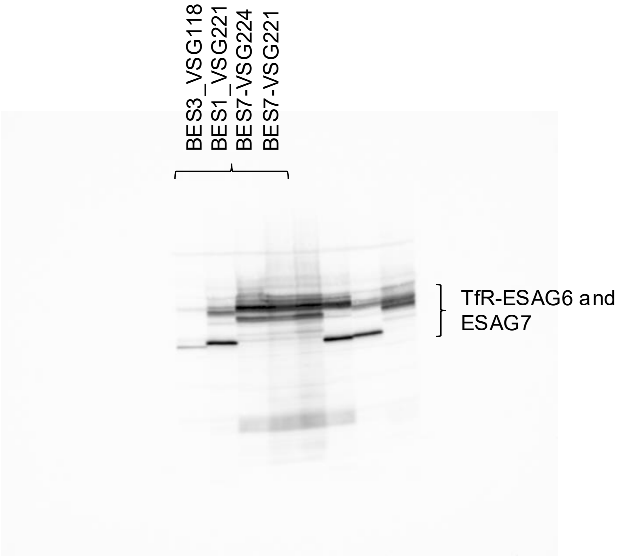

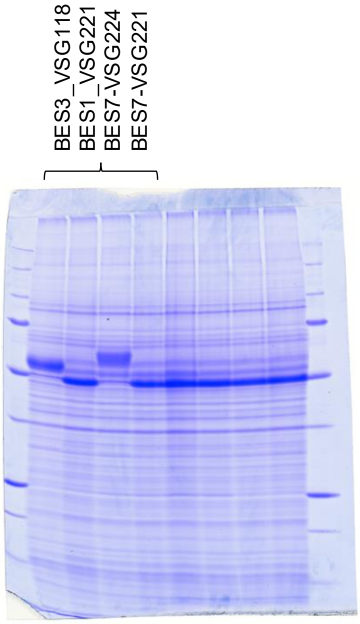

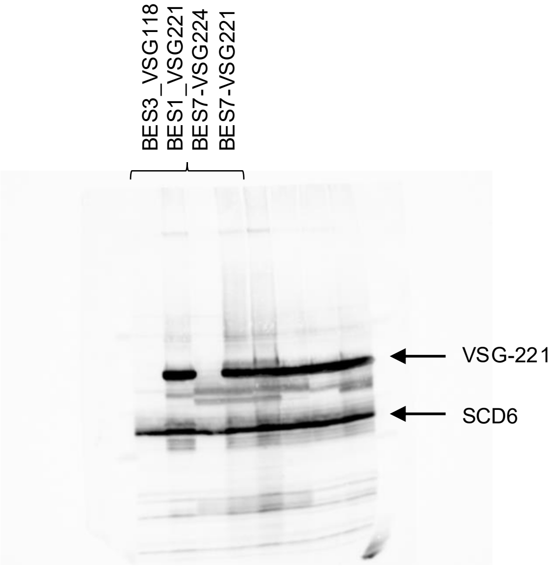

Figure 1, Source data 2. Original Coomassie Blue stained gel and membranes corresponding to Figure 1, panel B. Only the first four lanes are used in this figure. Markers are Precision Plus Protein Markers (BioRad): unstained for the Coomassie Blue gel and pre-stained all blue for the membranes. The same membrane was probed sequentially with anti-TfR antibodies then anti-SCD6 and anti-VSG221 antibodies.
