# Supplementary material for: Cell surface localisation of GPI-anchored receptors in Trypanosoma brucei
Source: eLife. 2026 May 19;14:RP107191. doi: 10.7554/eLife.107191 (PMC13186567; doi:10.7554/eLife.107191)
Supplement: Figure 2—source data 2. [file elife-107191-fig2-data2.zip › Figure 2_Source_data_2.pdf]

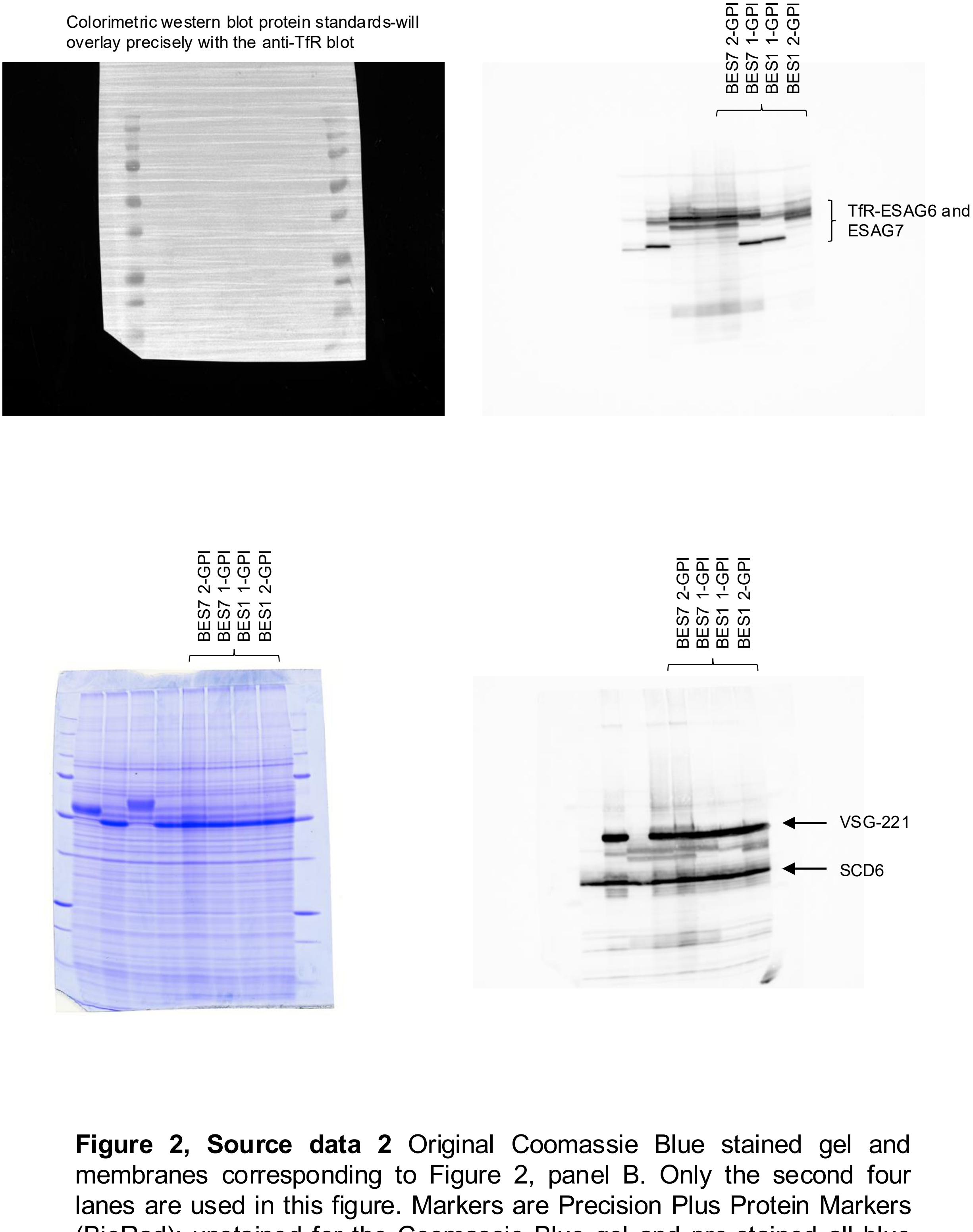

Figure 2, Source data 2 Original Coomassie Blue stained gel and membranes corresponding to Figure 2, panel B. Only the second four lanes are used in this figure. Markers are Precision Plus Protein Markers (BioRad): unstained for the Coomassie Blue gel and pre-stained all blue for the membranes. The same membrane was probed sequentially with anti-TfR antibodies then anti-SCD6 and anti-VSG221 antibodies.
