# Supplementary material for: Cell surface localisation of GPI-anchored receptors in Trypanosoma brucei
Source: eLife. 2026 May 19;14:RP107191. doi: 10.7554/eLife.107191 (PMC13186567; doi:10.7554/eLife.107191)
Supplement: Figure 3—figure supplement 4—source data 2. [file elife-107191-fig3-figsupp4-data2.zip › Figure S4_Source data 2.pdf]

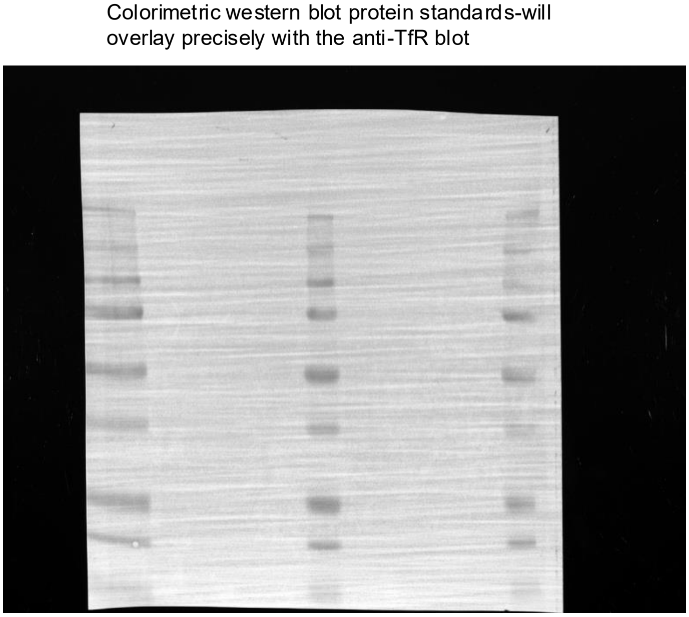

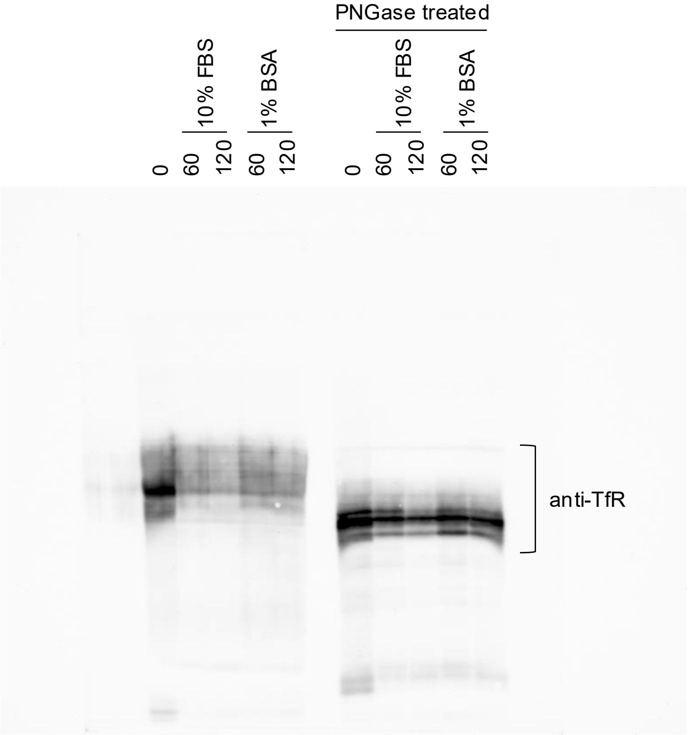

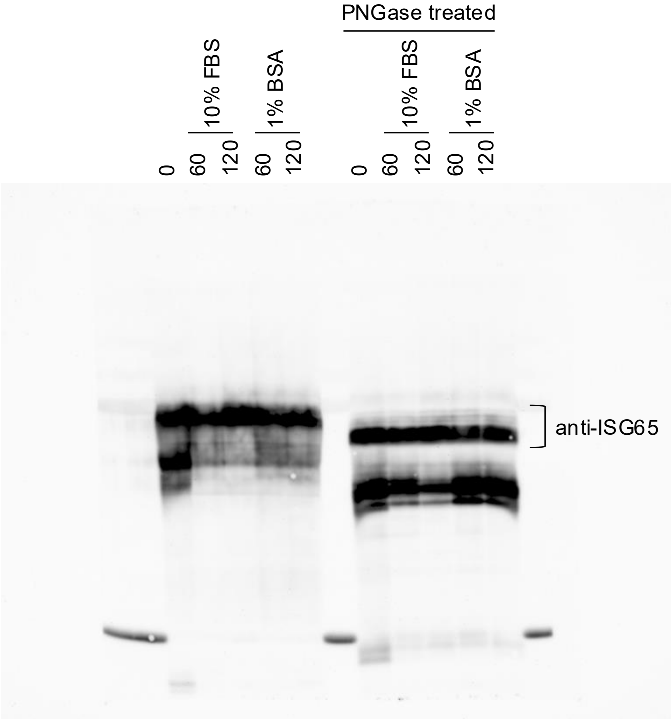

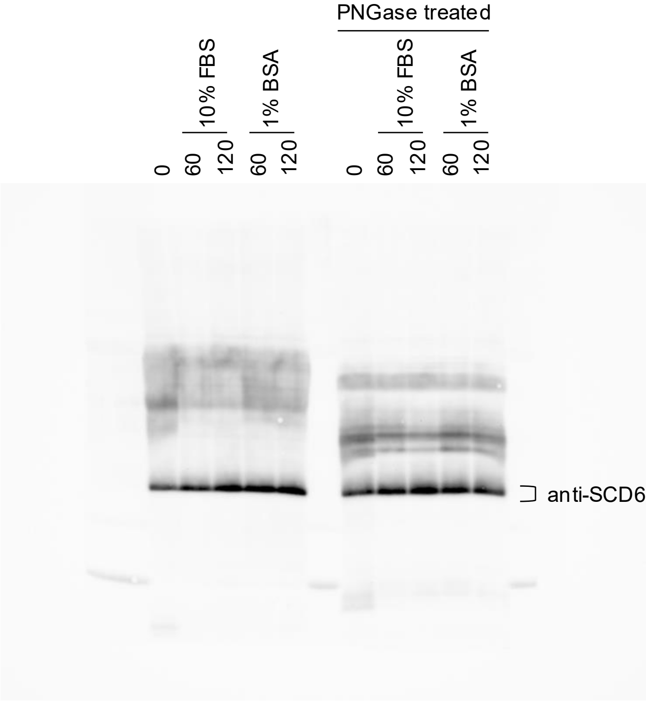

Figure S4, Source data 2. Original membranes corresponding to Figure S4, panel D. Markers are Pre-Stained all blue Precision Plus Protein Markers (BioRad). The same membrane was probed sequentially with anti-TfR, anti-ISG65 and anti-SCD6 antibodies.
